# Infrared and Harsh Light Visible Image Fusion Using an Environmental Light Perception Network

**DOI:** 10.3390/e26080696

**Published:** 2024-08-16

**Authors:** Aiyun Yan, Shang Gao, Zhenlin Lu, Shuowei Jin, Jingrong Chen

**Affiliations:** 1 College of Information Science and Engineering, Northeastern University, Shenyang 110167, China; yanaiyun@ise.neu.edu.cn (A.Y.); 2200813@stu.neu.edu.cn (S.G.); jinshuowei@ise.neu.edu.cn (S.J.); 2300800@stu.neu.edu.cn (J.C.); 2 Beijing Microelectronics Technology Institute, Beijing 100076, China

**Keywords:** image fusion, harsh light environment aware, information entropy, high-level vision tasks

## Abstract

The complementary combination of emphasizing target objects in infrared images and rich texture details in visible images can effectively enhance the information entropy of fused images, thereby providing substantial assistance for downstream composite high-level vision tasks, such as nighttime vehicle intelligent driving. However, mainstream fusion algorithms lack specific research on the contradiction between the low information entropy and high pixel intensity of visible images under harsh light nighttime road environments. As a result, fusion algorithms that perform well in normal conditions can only produce low information entropy fusion images similar to the information distribution of visible images under harsh light interference. In response to these problems, we designed an image fusion network resilient to harsh light environment interference, incorporating entropy and information theory principles to enhance robustness and information retention. Specifically, an edge feature extraction module was designed to extract key edge features of salient targets to optimize fusion information entropy. Additionally, a harsh light environment aware (HLEA) module was proposed to avoid the decrease in fusion image quality caused by the contradiction between low information entropy and high pixel intensity based on the information distribution characteristics of harsh light visible images. Finally, an edge-guided hierarchical fusion (EGHF) module was designed to achieve robust feature fusion, minimizing irrelevant noise entropy and maximizing useful information entropy. Extensive experiments demonstrate that, compared to other advanced algorithms, the method proposed fusion results contain more useful information and have significant advantages in high-level vision tasks under harsh nighttime lighting conditions.

## 1. Introduction

Images from different modalities obtained by infrared and visible sensors describe and interpret the imaging scene from different perspectives. The imaging results of visible sensors have abundant texture details and excellent visual effects, but they are sensitive to light intensity, while infrared sensors can ignore illumination variations to produce images with prominent targets, though with blurred details and susceptibility to noise. By integrating the key information of both modalities guided by entropy metrics and information theory, the fusion results maximize useful information and minimize noise, greatly enhancing the information entropy and completeness of the scene description in the fused images. Thus, the fusion of infrared and visible images, which is able to enhance scene visual effects and provide substantial assistance for subsequent high-level tasks, has been widely applied in areas such as intelligent vehicle assistance driving [1], road safety monitoring [2], and modern satellite remote sensing [3].

In visible–infrared image fusion research, numerous approaches have continually emerged. Successive improvement of methods has increased the effectiveness of fusion and demonstrated in high-level vision tasks. The methodologies for fusing infrared and visible images can be delineated into traditional methods [4] and deep learning approaches [5]. Traditional techniques are specifically categorized as methods based on sparse dictionary training [6], methods based on multi-scale transformations [7], methods based on low-rank representation learning [8], methods based on segmentation or saliency [9], and hybrid modeling methods [10]. In recent years, generalized neural network structures with good training results on huge datasets have been widely used for their excellent feature extraction capabilities that can be easily transformed into current fusion tasks to achieve superior fusion performance in contrast to traditional fusion algorithms. Deep learning methods based on deep learning are mainly categorized according to differences in network structures, such as autoencoder (AE) networks [11], generative adversarial networks (GANs) [12], convolutional neural networks (CNNs) [13], and transformers [14].

Although the current fusion results of deep neural networks are superior to traditional methods and overcome some drawbacks of traditional methods, such as the lack of targeted research on the distribution of visible image information under harsh light interference in scenarios like night roads, deep learning-based methods still struggle to achieve satisfactory results in both harsh light background and normal environments simultaneously. Specifically, first and foremost, most existing fusion methods are predicated on an antecedent assumption that the intensity of infrared image target pixels is prominent, and the gradients of visible images are replete with texture details, selectively extracting features from visible and infrared images. However, this assumption overlooks the importance of target boundary information in high-level vision tasks, such as object detection and semantic segmentation, leading to blurred target boundaries in fusion results, which impinge upon the efficacy of the fusion outcomes. Secondly, while mainstream advanced fusion methods can achieve good results in favorable environments, the contradiction between low information entropy and high pixel values in visible images under harsh light interference conditions results in significant target information being obscured by invalid harsh light information in the fused image, hindering the attainment of satisfactory fusion results. Finally, the majority of deep learning-based approaches leverage convolutional layers to extract a diverse array of features from images and subsequently feed them into the next level after rough concatenation. However, this approach overlooks the differences in feature levels, thereby diminishing the utilization of feature information. Consequently, during the fusion process, important features such as the target boundaries required for high-level vision tasks are partially lost, impinging upon the efficacy of fusion outcomes in ensuing high-level vision tasks. These challenges result in unique image fusion difficulties under nighttime harsh light conditions.

We designed a harsh light interference-resistant target edge information-guided feature fusion network to address the above issues. This network explores the potential of combining image information entropy and information distribution to guide fusion tasks, achieving high-performance fused images in both normal and harsh light environments, thus improving the outcomes of subsequent high-level vision tasks. The specific details of the network structure will be presented in Section 3, and the primary contributions of this manuscript are delineated as follows:

- An infrared salient target edge feature extraction module that includes high-level semantic interactive fusion (HSIF) and edge feature enhance block (EFEB) is designed, permitting the network to more precisely attend to salient semantic information at the edges, thus avoiding the blurring of the structure and edge details of salient targets in the fusion results.
- A harsh light environment aware (HLEA) module is designed. Based on the distribution of harsh light visible light image information, this module determines the feature fusion weights of the hierarchical feature fusion module to address the severe degradation of fusion performance under adverse lighting conditions due to low information entropy and high-intensity pixel value areas.
- An edge-guided hierarchical feature fusion (EGHF) module is designed. Combining target edge feature guidance with harsh light aware-weight allocation achieves efficient utilization of key semantic features under harsh light interference, avoiding the impact of blurred target boundaries and harsh light occlusion on ensuing high-level vision tasks in fusion results.

The residual portion of this manuscript is organized according to the following structure. In Section 2, relevant image fusion methods and key-related work are briefly described. Section 3 provides an in-depth discussion of our proposed fusion approach, covering aspects such as the network architecture, module design, and loss functions. In the subsequent section, we primarily elucidate the advantages of the fused images produced by our proposed network over those of other methods with regard to qualitative visual perception and quantitative nonparametric parameters under regular environments and challenging nighttime conditions with harsh light. In the final chapter of this paper, Section 5, we summarize the content of our research.

## 2. Related Work

In this chapter, we commence by surveying extant algorithms for the fusion of infrared and visible images. We then offer a concise overview of the relevant foundational knowledge involved in the harsh light environment-aware network proposed in this paper.

### 2.1. Traditional Image Fusion Methods

Traditional image fusion methodologies typically encompass three steps: image transformation, coefficient amalgamation, and image inverse transformation. In image fusion research, the most dynamic domain is centered on multi-scale transformation methods [15]. Multi-scale decomposition-based image fusion breaks down images into different scale representations, such as pyramids [16], wavelet transforms [17], etc. The early fusion approaches rooted in pyramid transformation were proposed by Burt et al. [18], which continuously filter the source image, with large scales at the bottom, gradually decreasing upwards, to obtain a structure similar to a pyramid, and after fusion, the final image with source image information is obtained, but the decomposition process is irreversible. In contrast, methods based on wavelet transforms can relatively easily capture both the structural features and intricate details from the source image and can effectively retain information throughout the decomposition process. Jose et al. [19] proposed an NSST multi-scale image fusion method that inherits the wavelet transform concept, decomposing images into sub-bands of different scales in multiple directions to capture features at various directional scales. This process avoids downsampling, thereby preventing resolution loss and better preserving image details. However, it faces challenges in expressing image boundaries and feature information. To address edge information retention, Shreyamsha et al. [20] introduced a fusion method called CBF. This method employs cross-bilateral filtering between source images to maintain the edge features of bimodal images while suppressing noise and other unnecessary details. Ma et al. [9] developed a gradient transfer method for image fusion. By computing gradients, extracting salient gradients, and integrating Poisson gradient fields, this method reconstructs fused images to retain more details and edge information. Zuo et al. [21] used filters that preserve edges, like guided filtering and bilateral filtering, to enrich the texture information of fusion images. In addition, Vaish et al. [22] used multi-resolution singular value decomposition (MSVD) technology to identify important and unimportant details, and the fundamental information was fused based on the absolute maximum value rule for critical information while employing sparse representation for less essential details, which is suitable for maintaining the intricate image details and edge delineation. Li et al. [23] designed a model using adaptive sparse representation. By learning a more compact dictionary to replace the initially redundant dictionary, pseudo shadows and distortions in the fusion image were effectively reduced while preserving image details and structures. Zhou et al. [24] presented a methodology for fusing infrared and visible images that employ the recognition of prominent objects, which guides the network to detect salient regions by introducing salient object masks, implicitly realizing salient object detection and key information fusion. While these traditional methods are generally simple and straightforward, their effectiveness is severely limited when tackling complex image fusion tasks.

### 2.2. Deep Learning Methods

A PCNN [25] is a pivotal aspect earliest proposed deep learning-based image fusion method, which utilizes deep learning models to acquire the feature representations and fusion rules of images through large-scale data training, enabling the learning of higher-level feature expressions from data and achieving end-to-end image fusion. Recently, deep learning-based advancements in image fusion methods have undergone rapid development, and according to the features and guiding rules of the algorithms, they can be classified into four distinct categories: approaches utilizing AE, methods based on GANs, methods based on CNNs, and transformer-based techniques.

Autoencoder-based methods typically involve three steps: first, pre-training the autoencoder with source images to extract their features; then, combining various source image features using fusion methods; and finally, reconstructing the images using the trained decoder. Prabhakar et al. [26] introduced an innovative unsupervised deep learning fusion architecture, named DeepFuse, designed for fusing static multiple-exposure images. However, the computation only utilized the output of the last layer, resulting in information loss. Addressing this, Li et al. [27] replaced the convolutions in DeepFuse with 3 × 3 convolutional kernels and improved the convolutional network by replacing it with a Dense-Block, proposing the groundbreaking DenseFuse network architecture. Building on the success of the DenseFuse dense connection structure, Li et al. [28] proposed NestFuse, which uses nested connections instead of the original dense connections to constrain the impact of semantic gaps. Additionally, NestFuse includes attention mechanism-based fusion rules to retain more effective information. Zhao et al. [29] were the first to propose an image fusion network where both decomposition and fusion are completed using AEs. The encoder decomposes the image into background feature maps and detail feature maps, which are then fused separately before being restored to the original image by the decoder. Leveraging the powerful feature extraction and representation capabilities of autoencoders, the generated fusion images exhibit significant improvements in detail and information content.

CNN-based methods offer unparalleled advantages in image feature extraction. The fundamental concept behind CNN-based methods is to train a CNN model to generate a fused outcome by training to discern the mapping relationship between input images. Li et al. [30] utilized a framework for deep learning to extract attributes from visible as well as infrared images. However, they encountered issues with the VGG-19 network’s structural deficiencies, resulting in the loss of certain valuable information during feature extraction. Addressing this, Li et al. [31] introduced a fusion architecture that utilizes zero-phase components along with deep features analysis as its foundation. Yang et al. [32] introduced a method for fusing visible and infrared images using latent low-rank representation in conjunction with convolutional neural networks, avoiding issues such as information loss, insufficient imaging quality, and complex structures. Xu et al. [33] introduced a method called DRF, which employs convolutional networks to perform disentangled representations for image fusion. The method decomposes the image information sources according to imaging principles, extracts scene and sensor modality feature representations via convolutional networks, and then fuses these representations using various strategies. Finally, the fused image is generated through a pre-trained generator. This method significantly improves the accuracy and interpretability of the fusion process.

GAN-based methods are an artificial intelligence algorithm used for generative modeling, consisting of a discriminator and a generator. The generator’s duty lies in producing synthetic images, whereas the discriminator assesses the genuineness or authenticity of those images. During training, the discriminator and generator engage in adversarial training, competing against each other, aiming to make the generated images as realistic as possible. Ma et al. [12] innovatively applied GANs to the infrared and visible image fusion method FusionGAN. Unlike traditional methods that depend on manually designed fusion rules, FusionGAN uses adversarial training to automatically learn how to fuse images, significantly reducing reliance on manual expertise. However, FusionGAN’s dependence on feedback solely from the discriminator may lead to issues with model convergence or unstable fusion image quality. GANMcC [34], which is based on FusionGAN, improves training stability by introducing multiple constraints, reducing the difficulty of convergence, and allowing the model to produce more consistent and reliable fusion results. Recently, GAN-based fusion methods have attracted more attention. Li et al. [35] introduced an unsupervised multi-domain image translation approach using structural consistency generative adversarial networks (SCGAN) to achieve fusion and transformation between images. Xu et al. [36] discussed the use of GANs for image fusion, including applications such as multi-modal image fusion and image restoration.

Transformer is an architecture that significantly improves the performance of deep learning translation models by using attention mechanisms. In 2021, Zhao et al. [37] applied a transformer to the image fusion discipline. Wang et al. [14] used a transformer-based encoder to extract global features and designed SwinFuse in an autoencoder-based framework.

Compared with traditional methods, deep learning could learn by extracting informative features from an extensive training dataset and has made significant strides in visual recognition, but it may reduce the quality of fusion under poor light interference, and the need for high-level vision tasks may be ignored.

### 2.3. Image Fusion Resilient to Interference

Some researchers have recognized that infrared and visible image fusion algorithms may drastically degrade the output quality under challenging adverse environmental conditions. Therefore, scholars have conducted targeted research on the interferences that may exist in fusion application environments, hoping to enhance the robustness of fusion networks and significantly bolster the performance of fusion results. Low-light scene image fusion initially received widespread attention [4]. Due to the low contrast and limited texture and color information fusion of visible images in low-light conditions, the visual effects of the fused images are unsatisfactory. Zhang et al. [38] designed an EV-Fusion network, which achieves the fusion of visible image information with infrared images through two stages of enhancing and fusing images, thus enhancing the image fusion effect in low-light environments. The EV-Fusion network considers the interference of low-light environments but neglects the interference of illumination changes on the fusion results. Considering this issue, Tang et al. [39] designed a sub-network for daytime and nighttime classification in PIAFusion. By calculating the weighting of the visible and infrared components in the loss function based on classification probabilities, differentiated network parameter updates were achieved for different illumination environments, thereby mitigating the influence of illumination changes on the outcomes of fusion. Furthermore, besides considering the interference of different illuminations, many researchers also consider uniformly considering all interferences to enhance the resistance of fusion networks to all possible disturbances that may degrade sensor imaging results. Luo et al. [40] introduced a fusion rule grounded on the richness of image information, aiming to preserve more meaningful content from input images, prevent highly prominent but contextually unimportant information in visible from overshadowing key information in infrared images, and achieve a hierarchical presentation of key information in images. Unfortunately, manually designed fusion rules are overly complex and poorly adapted to various actual interferences. Rao et al. [41] proposed an AT-GAN with an adverse environment estimation module. Using the evaluation results, the weights of the generator’s content loss function were automatically modified to complete network training. This approach enhances fusion effects in a more flexible and balanced manner, mitigating, to some extent, the impact of severe degradation on fusion outcomes under challenging conditions. However, the authors did not provide targeted ablation experiments in the article to showcase the efficacy of this design.

The above fusion networks designed to resist various interferences often fail to achieve high-quality fusion in harsh light environments due to the failure of the fusion rules. While some fusion methods have considered disturbances from different illuminations, such as low-light environment interference and illumination environment transformation interference, the absence of targeted research on harsh light environment interference also prohibits the achievement of fusion networks resistant to harsh light interference. Therefore, in our work, we propose a harsh light environment-aware module to achieve high-quality image fusion under challenging harsh light conditions. Specifically, we first estimate the illumination conditions of the environment and extract an illumination map using illumination classification and illumination decomposition sub-networks. We then determine the feature fusion weights in network fusion modules by the illumination probability and illumination map output by the sub-network and evaluate the quality level of the input images. This approach leads to the network incorporating more semantic information, rather than simple salient information, into the generated fused results, enhancing the scene interpretability and object recognizability, thus avoiding interference from irrelevant harsh light information.

## 3. Proposed Method

In this section, we initially present the overall structure of our network model. Then, we provide detailed explanations of the three core components of the network model: edge feature extraction, harsh light environment perception, and hierarchical feature fusion. Finally, we expound on the loss function used for network training.

### 3.1. Overview of Network Architecture

**Figure 1:**
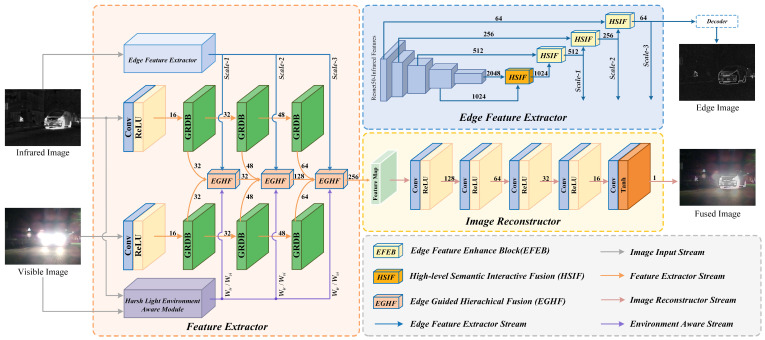
The architecture of the harsh light environment-aware visible and infrared image fusion network. The network backbone is composed of four components: multi-modal feature extraction, edge feature extraction, harsh light environment awareness, and image decoding and reconstruction.

To accomplish image fusion under harsh light interference, avoiding the occlusion of thermal target information by harsh light while improving the fusion efficacy results in subsequent high-level vision tasks, we designed a novel network for visible and infrared image fusion. The specific framework of the fusion network is depicted in Figure 1. The network backbone consists of four parts: multi-modal feature extraction, edge feature extraction, harsh light environment awareness, and image decoding and reconstruction.

*(1) Edge Feature Extraction:* Our model utilizes ResNet50 [42] pre-trained on the ImageNet dataset [43] for hierarchical preliminary feature extraction of infrared images. After removing the last part of the pooling layer and fully connected layer in the ResNet50 network, the remaining five convolutional groups are stacked as the backbone feature extractor. After passing through the backbone feature extractor, the features of five different levels output by the five convolutional groups are used as the preliminary feature representation of the infrared image. Additionally, the feature set extracted by the convolutional groups here is denoted as $E=\left\{E_{1},E_{2},E_{3},E_{4},E_{5}\right\}$. The features representing high-level semantic features, $E_{4}$ and $E_{5}$, are fused through HSIF to obtain semantic features representing the salient targets of the image, and then through the EFEB, different levels of shallow texture features, $E_{3}$, $E_{2}$, and $E_{1}$, are progressively guided through hierarchical forward feeding to obtain three different levels of salient target edge features.

*(2) Harsh Light Environment Awareness:* We designed a HLEA module to cope with background harsh light interference. First, the pre-trained illuminance classification sub-network and illuminance decomposition sub-network are used to predict the illuminance probability and illuminance map of visible images, and the infrared–visible feature weights are calculated by combining the two. Then, the weights are corrected using the Blind-Referenceless Image Spatial Quality Evaluator (BRI-SQUE) [44] to obtain the harsh light environment-aware weights. Here, the set of weights calculated by the HLEA is denoted as $W=\left\{W_{ir},W_{vi}\right\}$.

*(3) Image Feature Extraction:* We devised a dual-branch encoding structure for feature extraction from source images. In each branch, three gradient residual dense blocks (GRDBs) [45] are employed as the main components to extract fine-grained and deep features from the input images. Here, the feature sets obtained by the dual-branch extraction for visible and infrared images are represented as $F_{ir}=\left\{F_{ir}^{1},F_{ir}^{2},F_{ir}^{3}\right\}$ and $F_{vi}=\left\{F_{vi}^{1},F_{vi}^{2},F_{vi}^{3}\right\}$, respectively. In the process of merging features extracted from visible and infrared sources, instead of simple concatenation fusion, inspired by the self-attention mechanism, we used edge features $E_{i}$ to guide fusion features, enabling the embedding of prominent target edge features into the fusion features for subsequent transmission.

*(4) Image reconstruction:* For the features derived from the dual-branch encoder in visible and infrared fusion, we use a decoder to complete the image reconstruction. The decoder is a single-branch structure, consisting of five convolutional layers, with the first four utilizing the Relu activation function, and the final convolutional layer employing the Tanh activation function.

### 3.2. Edge Feature Extraction

Image fusion, serving high-level vision tasks, should aim to incorporate more semantic information into the fusion image to enhance subsequent tasks’ performance. However, as shown earlier [26,27,35], most fusion methods tend to focus on improving fusion result metrics, overlooking the importance of semantic information in the results for high-level vision. Specifically, the longstanding prior assumption that infrared images emphasize pixel intensity of significant objects while visible images have clear target texture details often leads to the neglect of edge texture features of infrared salient objects, which are crucial for high-level tasks. To address this, we have devised an edge feature extraction method tailored to infrared images that combines semantic and texture layer refinement, effectively extracting edge information of significant targets.

#### 3.2.1. High-Level Semantic Feature Interactive Fusion (HSIF)

**Figure 2:**
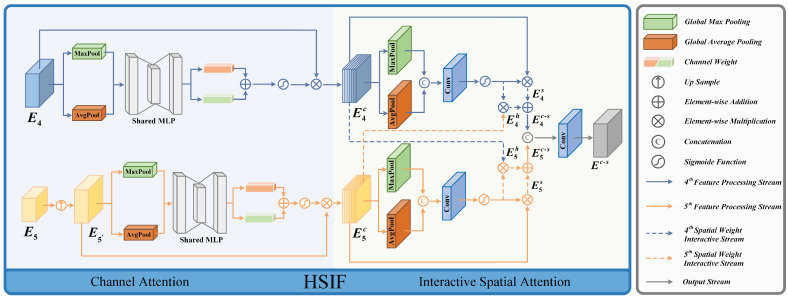
The architecture of the high-level semantic feature interactive fusion (HSIF) module. The channel attention mechanism reallocates channel weights for edge features *E*_4_ and *E*_5_ and then constructs an interactive spatial attention mechanism to obtain more comprehensive global semantic information.

Residual networks trained on extensive image datasets have been proven to have excellent feature extraction capabilities for images [46]. Therefore, we use ResNet50 to extract features from infrared images, obtaining features at different levels, where surface-level characteristics encapsulate intricate details about target edge textures, and deep features embody richer semantic content about target positions. For two consecutive deep features, their differences are much smaller than those between features from different hierarchical levels, indicating strong contextual semantic information. Therefore, we proposed the high-level semantic feature interactive fusion (HSIF) module to obtain semantic information about target positions in infrared images. The module’s detailed structure is depicted in Figure 2.

In HSIF, the input features $E_{4}\in R^{C_{4}\times H_{4}\times W_{4}}$ and $E_{5}\in R^{C_{5}\times H_{5}\times W_{5}}$, where $C_{i},\;H_{i},\;W_{i}$ represent the dimensions of feature $E_{i}$, including the number of channels, height, and width, respectively, and $H_{4}=2H_{5},W_{4}=2W_{5}$. First, we upsample feature $E_{5}$ to obtain feature $E_{5^{\prime }}$ with the same size as $E_{4}$ for subsequent spatial weight interaction. Then, we use attention methods to process $E_{4}$ and $E_{5^{\prime }}$ in the channel dimension; emphasis is placed on the pivotal feature channels. The specific process is described as follows:

(1) $$\begin{array}{l} E_{5^{\prime }}=Up(E_{4})\\ E_{i}^{c}=\sigma (MLP(GMP(E_{i})⊕GAP(E_{i})))⊗E_{i} \end{array}$$

where, $UP\left(\cdot \right)$ represents the upsampling operation, obtaining the feature $E_{5^{\prime }}\in R^{C_{5}\times H_{4}\times W_{4}}$. Moreover, “⊗” is element-wise multiplication, “⊕” is element-wise addition, $GMP\left(\cdot \right)$ is global max pooling, $GAP\left(\cdot \right)$ is global average pooling, $MLP\left(\cdot \right)$ is a multilayer perceptron, and $\sigma \left(\cdot \right)$ is the sigmoid normalization calculation. The obtained feature $E_{i}^{c}\in R^{C_{5}\times H_{4}\times W_{4}}$ has the same scale as the input feature.

Subsequently, in spatial attention processing, based on the similarity between high-level semantic features, we interactively process the feature information of $E_{4}$ and $E_{5}$ to obtain high-level features that have rich semantic information. Specifically, the spatial attention weights of features $E_{4}$ and $E_{5}$ are combined with neighboring features to achieve information interaction. The process is defined as follows:

(2) $$\begin{array}{l} E_{i}^{s}=\sigma (Conv(GMP(E_{i}^{c})⊕GAP{\left(E_{i}^{c}\right)}_{3})⊗E_{i}^{c},i=\{4,5\}\\ E_{4}^{h}=\sigma (Conv(GMP(E_{4}^{c})⊕GAP{\left(E_{4}^{c}\right)}_{3})⊗E_{5}^{c}\\ E_{5}^{h}=\sigma (Conv(GMP(E_{5}^{c})⊕GAP{\left(E_{5}^{c}\right)}_{3})⊗E_{4}^{c}\\ E^{c-s}=Conv{\left(Cat((E_{4}^{h}⊕E_{4}^{s}),(E_{5}^{h}⊕E_{5}^{s}))\right)}_{3} \end{array}$$

where $Conv{\left(\cdot \right)}_{3}$ is a 3×3 convolution operation, and $Cat\left(\cdot \right)$ is channel-wise concatenation operation. The processed feature $E_{i}^{s}\in R^{C_{i}\times H_{4}\times W_{4}}$ represents the feature after spatial attention processing, $E_{i}^{h}\in R^{C_{i}\times H_{4}\times W_{4}}$ represents the feature after information interaction, and $E^{c-s}\in R^{C_{4}\times H_{4}\times W_{4}}$ represents the prominent target position semantic feature obtained from HSIF, which can provide assistance for the extraction of texture edge features.

#### 3.2.2. Edge Feature Enhance Block (EFEB)

**Figure 3:**
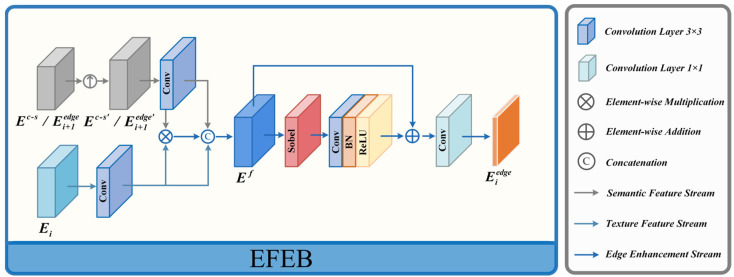
The architecture of the edge feature enhancement block (EFEB). Detail features are fused with deep features and then enhanced with the residual Sobel operator to strengthen the edges of salient objects.

The high-level features obtained after information interaction contain rich global semantic information, which can represent the contextual position relationship of prominent targets. However, how to better utilize semantic information to combine with shallow features to extract target edge features is still crucial. In previous fusion methods [24,36], high-level features are usually directly fed into shallow features at various levels to enrich the semantic information at different levels. However, this operation ignores the significant differences between different-level features, and the diversity between the features at different levels causes the deep features to not play their own important role. To address this issue, we designed the EFEB to pass cross-semantic information layer by layer to shallow features, reducing the problem of partial loss of edge features caused by feature differences. The module’s precise configuration is illustrated in Figure 3.

In the edge feature extraction module, there are three EFEB in total, where the third EFEB takes shallow edge texture features $E_{3}\in R^{C_{3}\times H_{3}\times W_{3}}$ and cross-deep semantic features $E^{c-s}$ as inputs, outputting feature $E_{3}^{edge}\in R^{C_{3}\times H_{3}\times W_{3}}$, while the first and second EFEBs take edge texture features $E_{2}\in R^{C_{2}\times H_{2}\times W_{2}}$ and $E_{1}\in R^{C_{1}\times H_{1}\times W_{1}}$ as inputs respectively, and the deep semantic features are the output features $E_{3}^{edge}\in R^{C_{3}\times H_{3}\times W_{3}}$ and $E_{2}^{edge}\in R^{C_{2}\times H_{2}\times W_{2}}$ from the previous EFEB module.

In EFEB, the input deep semantic features are initially upsampled to the same size as the shallow texture edge features. After extracting differential features by element-wise multiplication with the input shallow texture features and concatenating them with the two inputs, the Sobel operator with residual structure is applied to strengthen edges, resulting in texture edge features of significant targets at different levels of the image. The specific implementation processes of the first and second EFEB modules are as follows:

(3) $$\begin{array}{l} E_{i+1}^{edge^{\prime }}=Up(E_{i+1}^{edge})\\ E^{f}=Cat(E_{i+1}^{edge^{\prime }},E_{i},E_{i+1}^{edge^{\prime }}⊗E_{i})\\ E_{i}^{edge}=Conv_{1}(CBR(s(E^{f}))⊕E^{f})\;i=\{1,2\} \end{array}$$

where $Conv{\left(\cdot \right)}_{1}$ denotes a 1 × 1 convolution operation,$\;CBR\left(\cdot \right)$ represents convolutional, normalization, and activation function layers, with convolutional layers being 3 × 3 convolutions, and s(·) is the Sobel operator. $E_{i+1}^{edge}\in R^{C_{i+1}\times H_{i+1}\times W_{i+1}}$ represents the input deep semantic features, which are also the output of the previous EFEB module. $E_{i+1}^{edge^{\prime }}\in R^{C_{i+1}\times H_{i}\times W_{i}}$ stands for the upsampled deep semantic features, $E^{i}\in R^{C_{i}\times H_{i}\times W_{i}}$ represents the input texture edge features, $E^{f}\in R^{C_{f}\times H_{i}\times W_{i}}$ denotes the features after combining texture edges with semantic position, and $E_{i}^{edge}\in R^{C_{i}\times H_{i}\times W_{i}}$ signifies the enhanced significant target edge features output by the module.

The detailed implementation procedure of the third EFEB module unfolds as follows:

(4) $$\begin{array}{l} E^{c-s^{\prime }}=Up(E^{c-s})\\ E^{f}=Cat(E^{c-s^{\prime }},E_{3},E^{c-s^{\prime }}⊗E_{3})\\ E_{3}^{edge}=Conv_{1}(CBR(s(E^{f}))⊕E^{f}) \end{array}$$

where $E^{c-s^{\prime }}\in R^{C_{4}\times H_{3}\times W_{3}}$ represents the result obtained after upsampling the output of the HSIF module, and $E_{3}^{edge}\in R^{C_{3}\times H_{3}\times W_{3}}$ denotes the resulting features of the third EFEB module. In contrast to the first and second EFEB modules, its deep features originate from the HSIF module rather than other EFEB modules. Ultimately, the three EFEB modules obtain edge semantic features at three hierarchical levels, $E_{i}^{edge}\in R^{C_{i}\times H_{i}\times W_{i}}$, where *i* = {1, 2, 3}.

### 3.3. Harsh Light Environment Awareness

**Figure 4:**
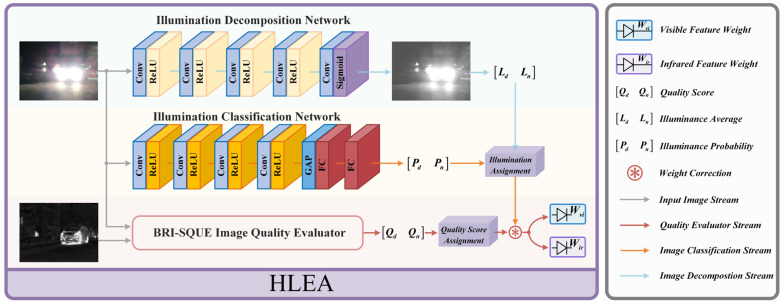
The architecture of the harsh light environment aware (HLEA) module. The module completes the prediction of the ambient light intensity level for input infrared and visible images through three parts: illuminance decomposition sub-network, illuminance classification sub-network, and image quality assessment.

Improving the anti-interference capability of fusion networks has always been a focal point of fusion algorithm research. As mentioned earlier, some fusion methods [39,40,41] have made targeted improvements to their robustness against low-light conditions, smoke occlusion, and image degradation interference. However, there is a lack of targeted research on enhancing the algorithm’s resistance to harsh light interference, which is commonly encountered in scenes like nighttime roads. This lack of research leads to a significant deterioration in the fusion image quality under harsh light interference for most fusion methods. Some algorithms that exhibit some resistance to harsh light environment interference are unable to provide a reasonable explanation for their ability to resist harsh light interference due to the lack of targeted research and do not offer corresponding ablation experiments. Consequently, the anti-interference performance of these algorithms fails to provide substantial theoretical assistance for the research of other fusion networks resistant to harsh light interference, thereby greatly restricting the study of fusion network resistance to harsh light interference and subsequent method improvements. To address this issue and achieve a high-quality fusion of images under harsh light interference, thereby providing a theoretical foundation for subsequent research on networks resistant to harsh light interference, we conducted targeted research on harsh light interference and proposed a harsh light environment aware (HLEA) module. The module’s intricate configuration is illustrated in Figure 4.

HLEA comprises an illuminance classification network [39], an illuminance decomposition network [47], and BRI-SQUE. Inspired by progressive illuminance-aware fusion networks [39], the distribution of information in visible and infrared images varies under different lighting conditions. Specifically, the main information in daytime scenes can be reflected in visible images, while nighttime infrared images harbor a wealth of pertinent information. Therefore, we utilize the illuminance classification network to estimate the probability of visible images being classified as daytime or nighttime categories, enabling fusion results under different scenes to selectively incorporate more pertinent information from either infrared or visible images. The specific implementation procedure unfolds as follows:

(5) $$\left[P_{d},P_{n}]=N_{IC}(I_{vi}\right)$$

where $P_{d}$ and $P_{n}$ represent the probabilities of the scene belonging to daytime or nighttime, respectively, $N_{IC}$ is the illuminance classification network, and $I_{vi}$ is the input visible image.

Solely relying on the $N_{IC}$ to compute perceptual weights has its limitations. Under harsh light conditions, the overall brightness of visible images is high, leading to an increased bias in prediction probabilities. To address this issue, we introduce the illuminance decomposition network. This network disassembles the input image into an illuminance map and detail map, where the illuminance map effectively reflects the brightness distribution of the visible image. Given the harsh light background, the prediction of $P_{d}$ by $N_{IC}$ tends to be higher. At this time, the illuminance map also exhibits a higher average value for the first 50% of pixels. Therefore, we use the pixel mean of the illuminance map to correct the perceptual weights calculated from the predictions of the $N_{IC}$. Consequently, the corrected perceptual weight of infrared features increases, while that of visible features decreases. This enhances the fusion of infrared thermal target information and reduces the fusion of meaningless harsh light information in visible images, thereby enhancing the effectiveness of image fusion under harsh light conditions. The specific process is as follows:

(6) $$\begin{array}{l} L=N_{ID}(I_{vi}),L_{day}=\frac{P_{d}}{L_{pixf}},L_{night}=\frac{P_{n}}{L_{pixl}}\\ W_{vi}^{c^{\prime }}=\frac{L_{day}\cdot P_{d}}{\left(L_{day}+L_{night})(P_{d}+P_{n}\right)},W_{ir}^{c^{\prime }}=\frac{L_{night}\cdot P_{n}}{\left(L_{day}+L_{night})(P_{d}+P_{n}\right)}\\ W_{vi}^{c}=\frac{W_{vi}^{c^{\prime }}}{W_{vi}^{c^{\prime }}+W_{ir}^{c^{\prime }}},W_{ir}^{c}=\frac{W_{ir}^{c^{\prime }}}{W_{vi}^{c^{\prime }}+W_{ir}^{c^{\prime }}} \end{array}$$

where L represents the illuminance map of the visible image, $N_{ID}$ is the illuminance decomposition network, $L_{pixf}$ and $L_{pixl}$ represent the grayscale mean of the first 50% and the last 50% pixels of the illuminance map, $L_{day}$ and $L_{night}$ are the daytime and nighttime correction coefficients of the illuminance map, and $W_{vi}^{c}$ and $W_{ir}^{c}$ denote the corrected perceptual weights of visible and infrared after illuminance map correction.

In the third part, considering the different degrees of degradation of visible and infrared images under harsh light interference, we use the image quality assessment algorithm BRI-SQUE to evaluate the quality of the source images and calculate the perceptual weights based on the evaluation quality scores. The specific calculation process is as follows:

(7) $$W_{vi}^{q}=\frac{Q_{ir}}{Q_{vi}+Q_{ir}},W_{ir}^{q}=\frac{Q_{vi}}{Q_{vi}+Q_{ir}}$$

where $Q_{vi}$ and $Q_{ir}$ represent the visible and infrared image quality scores computed using the parameter-free quality assessment algorithm BRI-SQUE. A higher score reflects worse image quality, while a lower score indicates better image quality, respectively. $W_{vi}^{q}$ and $W_{ir}^{q}$ represent the perceptual weights of visible and infrared data computed from the image quality scores.

Finally, the perceptual weights are calculated with $N_{IC}$ and $N_{ID}$ are corrected using BRI-SQUE-calculated perceptual weights to obtain the final result. The specific correction process is as follows:

(8) $$\begin{array}{l} W_{ir}=(1-a)W_{ir}^{c}+aW_{ir}^{q}\\ W_{vi}=(1-a)W_{vi}^{c}+aW_{vi}^{q} \end{array}$$

where $W_{ir}$ and $W_{vi}$ are the perceptual weights of infrared as well as visible, respectively, output by the HLEA module. Notably, in this context, a is a correction coefficient used to balance the weights obtained from the illumination assessment and the weights calculated with the image quality score evaluator. In our experiments, we empirically set it to 0.2 to balance these two factors.

### 3.4. Edge Guided Hierarchical Fusion

**Figure 5:**
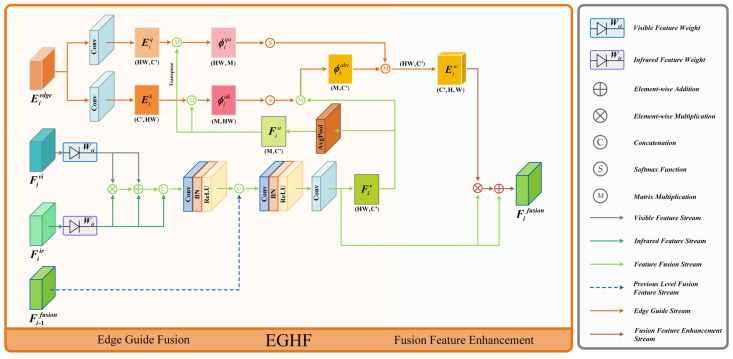
The architecture of the edge-guided hierarchical fusion (EGHF) module. In the edge guidance part, the infrared and visible features are fused according to the perceptual weights and then complete the redistribution of attention weights under the guidance of edge features. In the feature enhancement part, the infrared and visible features are added to the features guided by edges to achieve feature enhancement.

After obtaining the edge features of salient objects through the edge feature extraction module, directly concatenating or element-wise adding the edge features with the infrared–visible feature channels to integrate features can easily lead to edge noise or redundant information interference, making it difficult to fully utilize the edge features. To address this issue and enhance the guiding significance of salient object edge features, we propose an edge-guided hierarchical fusion (EGHF) module to handle the interaction and fusion of infrared–visible features and salient object edge features at different levels. Instead of directly concatenating or adding the edge features to the infrared–visible features for fusion, the edge features are used as guidance information for the infrared–visible features, achieving comprehensive feature fusion. The module’s detailed arrangement is presented in Figure 5.

Inspired by the multi-head self-attention mechanism in transformers [48], which captures long-range dependencies effectively, we capture the correlation between edge features and infrared–visible features and, based on this, complete the fusion guided by edge features. Specifically, we first upsample the edge features $E_{i}^{edge}\in R^{C_{i}\times H_{i}\times W_{i}}$ to $E_{i}^{edge^{\prime }}\in R^{C_{i}\times H\times W}$ to match the scale of the visible–infrared features, where H and W represent the dimensions of the input image, including the height and width, and the infrared and visible features at each level, respectively. Then, we obtain query matrices $E_{i}^{q}\in R^{HW\times C^{\prime }}$ and key matrices $E_{i}^{k}\in R^{C^{\prime }\times HW}$ through linear projection by convolutional layers, where $C^{\prime }=2C_{i}$. The specific process is as follows.

(9) $$\begin{array}{l} E_{i}^{edge^{\prime }}=Up(E_{i}^{edge})\\ E_{i}^{q}=v_{1}(Conv_{1}(E_{i}^{edge^{\prime }}))\\ E_{i}^{k}=v_{2}(Conv_{1}(E_{i}^{edge^{\prime }})) \end{array}$$

where $v_{1}\left(\cdot \right)$ denotes the transformation operator that converts the matrix dimension from $R^{A_{1}\times A_{2}\times A_{3}}$ to $R^{A_{2}A_{3}\times A_{1}}$, and $v_{2}\left(\cdot \right)$ represents the transformation operator that converts the matrix dimension from $R^{A_{1}\times A_{2}\times A_{3}}$ to $R^{A_{1}\times A_{2}A_{3}}$, $E_{i}^{edge}$ denotes the edge features outputted by the i-th EFEB module, $E_{i}^{edge^{\prime }}$ represents the upsampled edge features, and $E_{i}^{q}$ and $E_{i}^{k}$ denote the query matrices and key matrices corresponding to the edge features.

After obtaining the query matrices $E_{i}^{q}$ and key matrices $E_{i}^{k}$ corresponding to the edge features, establishing a correlation between the edge features and the infrared–visible features, value matrices need to be extracted from the infrared–visible features. Firstly, the fusion of infrared features and visible features is performed using the perception weights $W_{ir}$ and $W_{vi}$ obtained from the HLEA module to control the feature input ratio for anti-harsh light interference fusion. The processed perception weights for infrared and visible features are $F_{i}^{ir^{\prime }}\in R^{C\times H\times W}$ and $F_{i}^{vi^{\prime }}\in R^{C\times H\times W}$, respectively. Then, the differences and common features are highlighted separately by element-wise multiplication and addition of the two modalities. Finally, after concatenating the results of multiplication and addition with the input feature channels, the fused feature $F_{i}^{ir-vi}\in R^{HW\times C^{\prime }}$ is obtained through the $CBR\left(\cdot \right)$ layer. After obtaining the infrared–visible fusion feature, a value matrix $F_{i}^{v}\in R^{HW\times C^{\prime }}$ is obtained through linear projection by a convolutional layer. It is noteworthy that during the fusion process of visible and infrared features at the second and third levels, the fusion features $F_{i-1}^{fusion}\in R^{C\times H\times W}$ from the previous layer is introduced. Therefore, there are differences in feature input between the fusion modules EGHF of the second and third levels and the EGHF of the first level. The process of deriving the value matrices for the fused features at the initial level proceeds as follows:

(10) $$\begin{array}{l} F_{1}^{ir^{\prime }}=W_{ir}\cdot F_{1}^{ir},F_{1}^{vi^{\prime }}=W_{vi}\cdot F_{1}^{vi}\\ F_{1}^{ir-vi}=CBR(CBR(Cat(((F_{1}^{ir^{\prime }}⊗F_{1}^{vi^{\prime }})⊕F_{1}^{ir^{\prime }}⊕F_{1}^{vi^{\prime }}),F_{1}^{ir^{\prime }})))\\ F_{1}^{v}=v_{1}(Conv_{1}(F_{1}^{ir-vi})) \end{array}$$

where $F_{1}^{ir}$ and $F_{1}^{vi}$ denote the infrared and visible features of the first level, while $F_{1}^{ir^{\prime }}$ and $F_{1}^{vi^{\prime }}$ represent the infrared and visible features after perception weight processing. $F_{1}^{ir-vi}$ stands for the integrated feature after merging the infrared and visible images, and $F_{1}^{v}$ corresponds to the value matrix of the infrared–visible features.

In the integration of infrared and visible features in the second and third levels, a distinction from the first level is the concatenation of the fusion output $F_{i-1}^{fusion}$ from the previous level after the first $CBR\left(\cdot \right)$ layer and before the second $CBR\left(\cdot \right)$ layer. The detailed process is illustrated below:

(11) $$\begin{array}{l} F_{i}^{ir-vi}=CBR(Cat(CBR(Cat(((F_{i}^{ir^{\prime }}⊗F_{i}^{vi^{\prime }})⊕F_{i}^{ir^{\prime }}⊕F_{i}^{vi^{\prime }}),F_{i}^{ir^{\prime }}))))\\ i=\{2,3\} \end{array}$$

where $F_{i}^{ir^{\prime }}$ and $F_{i}^{vi^{\prime }},$ respectively, represent the infrared and visible features of the i-th level after perceptual weight processing, and $F_{1}^{ir-vi}$ represents the integrated features of infrared and visible images at the ith level, where i takes the values of 2 and 3, corresponding to the second and third levels.

Upon obtaining the query matrices $E_{i}^{q}$, key matrices $E_{i}^{k}$, and value matrices $F_{i}^{v}$, the calculation method of edge attention-guided weight matrix values follows the self-attention mechanism in the transformer. However, this approach results in attention weights of size $\left(HW,HW\right)$ when computing through matrix multiplication of $E_{i}^{q}$ and $E_{i}^{k}$. For example, for a 640×480 input image, the attention weight size is $\left(307,200,307,200\right)$, which is impractical for hardware computing resources. To address this issue, inspired by the Agent attention mechanism [49], we introduce an intermediate quantity $F_{i}^{a}$ to reduce computational complexity. The specific calculation process is as follows:

(12) $$\begin{array}{l} F_{i}^{a}=GAP(F_{i}^{ir-vi})\\ \phi_{i}^{qa}=mul(E_{i}^{q},T(F_{i}^{a})),\phi_{i}^{ak}=mul(F_{i}^{a},E_{i}^{k})\\ \phi_{i}^{akv}=mul(\phi_{i}^{ak},F_{i}^{v})\\ E_{i}^{w}=v_{3}(mul(\phi_{i}^{qa},\phi_{i}^{akv})) \end{array}$$

where $F_{i}^{a}\in R^{M\times C^{\prime }}$ represents the intermediate quantity obtained after global average pooling of infrared–visible features, $mul\left(\cdot \right)$ denotes matrix multiplication, $T\left(\cdot \right)$ denotes the matrix transpose operator, $\phi_{i}^{qa}\in R^{HW\times M}$ represents the intermediate query matrix, $\phi_{i}^{ak}\in R^{M\times HW}$ represents the intermediate key matrix, $\phi_{i}^{akv}\in R^{M\times C^{\prime }}$ represents the intermediate weight matrix, $v_{3}\left(\cdot \right)$ represents the operation of transforming matrix dimensions from $R^{A_{1}A_{2}\times A_{3}}$ to $R^{A_{3}\times A_{1}\times A_{2}}$, and $E_{i}^{w}\in R^{C^{\prime }\times H\times W}$ denotes the edge feature weight matrix. Introducing intermediate variables breaks down the process of computing the weight matrix into multiple steps, transforming space complexity into time complexity, thereby avoiding the unacceptable computational complexity of hardware resources.

Upon establishing the correlation between edge features and infrared–visible features, the attention weight matrix of edge features is obtained. Using this matrix, weights are allocated to the infrared–visible features, realizing feature fusion guided by edge features. The detailed process is outlined below:

(13) $$\begin{array}{l} F_{i}^{fusion}=(E_{i}^{w}⊗F_{i}^{ir-vi})⊕F_{i}^{ir-vi}\\ i=\{1,2,3\} \end{array}$$

where $F_{i}^{fusion}\in R^{C\times H\times W}$ represents the output fusion feature of the EGHF module at the i-th level, where *i* takes values of 1, 2, and 3, each representing different levels of feature fusion.

### 3.5. Loss Function

To enhance the network’s performance regarding qualitative visual perception and quantitative non-reference estimation indicators and to obtain high-quality fusion images under harsh lighting conditions, our model selects content loss and semantic loss to form a joint loss function, which is stated in the following manner:

(14) $$L_{fusion}=L_{con}+\lambda L_{sem}$$

where $L_{con}$ signifies the content loss, $L_{sem}$ signifies the semantic loss, and λ represents the semantic loss guidance level of the fusion network training. The content loss $L_{con}$ consists of the following two components:

(15) $$L_{con}=L_{int}+\alpha L_{texture}$$

where $L_{int}$ is the pixel intensity loss, which reflects the disparity in pixel values from the network input to the final output, and $L_{texture}$ is the detail texture loss, which reflects the difference from the network input to the final output in the texture detail. α is the equilibrium factor. The pixel intensity loss $L_{int}$ is formulated as follows:

(16) $$L_{int}=\frac{1}{HW}{∥I_{fused}-max(I_{vis},I_{ir})∥}_{1}$$

where H is the image height, W is the image width, $\|\cdot {\|}_{1}$ is calculated as a norm of 1, $I_{fused}$ is the pixel intensity value of the fused output source, $I_{ir}$ is the pixel intensity value of the infrared light source, and the $I_{vis}$ is the pixel intensity value of the visible source.

In addition, in order to highlight salient targets while preserving additional meaningful texture structure information, we define the detail texture loss $L_{texture}$ as follows:

(17) $$L_{texture}=\frac{1}{HW}{\left\|\left|\nabla I_{fused}|-max(|\nabla I_{vis}|,|\nabla I_{ir}|\right)\right\|}_{1}$$

where $|\left|\cdot \right|{|}_{1}$ represents calculated as a norm of 1, $\left|\cdot \right|$ represents the absolute value operation, and ∇ represents the Sobel gradient operator of the image texture.

In order to enhance the comprehensibility of the scene in the fusion result, we introduce the following semantic segmentation network and semantic loss: [45]

(18) $$\begin{array}{l} L_{sem}=L_{sem\_main}+\beta L_{sem\_aux}\\ L_{sem\_main}=\frac{-1}{HW}\sum_{c=1}^{C}\sum_{h=1}^{H}\sum_{w=1}^{W}L_{so}^{\left(c,h,w\right)}log(I_{sem}^{\left(c,h,w\right)})\\ L_{sem\_aux}=\frac{-1}{HW}\sum_{c=1}^{C}\sum_{h=1}^{H}\sum_{w=1}^{W}L_{so}^{\left(c,h,w\right)}log(I_{sem\_a}^{\left(c,h,w\right)}) \end{array}$$

Among them, the categories of *C* semantic segmentation, $I_{sem}\in R^{C\times H\times W}$ and $I_{sem\_a}\in R^{C\times H\times W}$ are the segmentation results and auxiliary segmentation results of the segmentation network output, respectively, and $\;L_{s}\in {\left(1,C\right)}^{H\times W}$ represents the result representation obtained from the single hot-coded label. The use of semantic loss can not only segment networks but also better assist the training of fusion networks.

## 4. Experiments

In this segment, to illustrate the superiority and advancement of our proposed fusion approach, we performed experiments to compare different approaches on multiple infrared and visible datasets and module ablation experiments. First, we presented the specifics of the experimental configurations and selection of evaluation metrics. Second, we compared our proposed fusion method with nine mainstream fusion methods in fusion tasks and high-level vision detection task, including three traditional methods, namely the following: CBF [20], NSST [19], and GTF [9], two AE-based methods, namely DIDFuse [29] and NestFuse [28], two GAN-based methods, namely FusionGAN [12] and GANMcC [34], and two CNN-based methods, namely PIAFusion [39] and DRF [33]. Finally, we performed ablation experiments to validate the efficacy of the module design within our proposed methodology.

### 4.1. Experimental Details

#### 4.1.1. Cooperative Training Strategy

For our proposed network model, we selected the MSRS [45] dataset, which was filtered and processed from the MFNet [50] dataset, for training. In the MSRS dataset, there are a total of 1083 training data comprising pairs of visible and infrared images. To prevent overfitting due to the small sample size, we applied data augmentation methods like random cropping, color jittering, random scaling, and horizontal flipping to original training samples. Additionally, we incorporated learning rate warm-up, learning rate decay, and the Adam optimization strategy to prevent training divergence due to inappropriate learning rates.

To include richer semantic information in the fused images, we have introduced a segmentation network and added segmentation semantic loss to the fusion loss. Therefore, designing a cooperative training algorithm for the fusion and segmentation networks to coordinate the training of both tasks becomes essential. For this reason, we have devised a cooperative training strategy as shown in Algorithm 1.
**Algorithm 1**: Adaptive Cooperative Training Strategy**Network Input:** Visible image $I_{vi}$ and infrared image $I_{ir}$
**Network Output:** Fused image $I_{fused}$Select a visible images and labels $\left\{\left(I_{vi}^{1},L_{vi}^{1}\right),\ldots \left(I_{vi}^{a},L_{vi}^{a}\right)\right\}$;
Train illumination classification network $N_{IC}$with the Adam optimizer: $\nabla_{N_{IC}}\left\{L_{class}\left(N_{IC}\right)\right\}$;
Select b visible images $\left\{I_{vi}^{1},I_{vi}^{2}\ldots I_{vi}^{b}\right\}$;
Train illumination decomposition network $N_{IS}$ by the Adam optimizer: $\nabla_{N_{ID}}\left\{L_{split}\left(N_{ID}\right)\right\}$;
**for**
m ≤ Max *iterations*
M
**do**
**for** p
*steps* **do**
Select c visible images $\left\{I_{vi}^{1},I_{vi}^{2}\ldots I_{vi}^{c}\right\}$;
Select c infrared images $\left\{I_{ir}^{1},I_{ir}^{2}\ldots I_{ir}^{c}\right\}$;
Update the balancing parameter λ according to $\lambda =\tau \left(1+e^{-\left(m-1\right)}\right)$
Update the weight parameters of fusion network $N_{f}$ with the Adam optimizer: $\nabla_{N_{f}}\left\{L_{fusion}\left(N_{f}\right)\right\};$
**end**
Utilize the fusion network to generate fused images for the subsequent segmentation network training;
**for** q
*steps* **do**
Selecting d fused images $\left\{I_{f}^{1},I_{f}^{2}\ldots I_{f}^{d}\right\}$ from the output of the fusion network;
Update the weight parameters of segmentation network $N_{s}$ with the Adam optimizer: $\nabla_{N_{s}}\left\{L_{sem}\left(N_{s}\right)\right\}$;
**end**


In the training process of Algorithm 1, we first complete the pre-training of the illumination classification and decomposition sub-networks in the HLEA module before moving on to the cooperative training of the fusion and segmentation networks. These two processes are relatively independent. In one phase of the joint training, we first train the fusion network to obtain fused images, then pass these images into the segmentation network to train and obtain segmentation results. These results guide the subsequent training phase of the fusion network. Through multi-phase training, the fusion network can produce fused images that achieve better segmentation results, containing richer semantic information. It is noteworthy that the guidance provided by the segmentation network to the fusion network varies at different stages. In the initial stage, the segmentation results cannot accurately reflect the richness of semantic information in the fused images, so the segmentation network should have less influence on the fusion process. As training progresses, the accuracy of the segmentation results improves, and its influence on the fusion process should gradually deepen. This is achieved by adjusting the balancing parameter λ in the fusion loss $L_{fusion}$.

In Algorithm 1, $N_{IC}$, $N_{ID}$, $N_{f}$, and $N_{s}$ are the illumination classification sub-network, illumination decomposition sub-network, image fusion network, and semantic segmentation network, respectively. $L_{class}$, $L_{split}$, $L_{fusion}$, and $L_{sem}$ represent the illumination classification loss function, illumination decomposition loss function, fusion loss function, and semantic segmentation loss function, respectively. m denotes the current training stage, M is the maximum training stage set to M = 4, p is the maximum number of iterations for the fusion network set to p=2700, and q represents the maximum number of iterations for the segmentation network set to q=20000. $\lambda =\tau \left(1+e^{-\left(m-1\right)}\right)$ is the variation function of the semantic loss balance parameter λ, with τ set to 3.15. As the training progresses, the accuracy of the segmentation network gradually increases, enhancing the guiding significance of the semantic loss, so λ should increase gradually. Given the non-linear improvement of network performance during training, the rate of increase of λ should slow down, which is consistent with the designed variation function of the balance parameter λ.

Furthermore, we set the texture loss coefficient to α=10 and the auxiliary semantic loss coefficient to β=0.1. Finally, we implemented the model on the PyTorch platform and optimized our proposed fusion algorithm using the Adam optimizer based on the designed loss functions. Additionally, all experiments were conducted using NVIDIA-RTX4080 graphics processing units (GPUs) and Intel i9-13900K central processing units (CPUs).

#### 4.1.2. Fusion Evaluation Metrics

Quantitative evaluation of fusion results can be approached from the perspective of the fusion task as well as high-level vision tasks. Specifically, for the fusion task, quantitative evaluation of fused image quality usually requires the use of no-reference metrics. In our work, we used EN [51], $FMI_{pixel}$ [52], SD [53], $Q_{abf}$ [54], MS-SSIM [55], VIF [56], and PSNR [57] to quantitatively evaluate our model and comparative methods on the TNO [58], MFNet, and M3FD [59] datasets. These metrics evaluate image quality from various perspectives. EN and $FMI_{pixel}$ evaluate from an information theory perspective. EN measures the information content in the fused image, and $FMI_{pixel}$ assesses the mutual information between the fused and original images at the pixel level. MS-SSIM and $Q_{abf}$ evaluate from the perspective of structural similarity between the source and fused images. MS-SSIM measures the consistency of multi-scale structures, while $Q_{abf}$ assesses the preservation of information in the fused image. SD and PSNR are evaluated from the perspective of image characteristics. SD represents the standard deviation of the image, indicating its contrast, while PSNR quantifies the difference between the fused and source images, used to assess image reconstruction quality. VIF assesses from a human perception-inspired perspective, estimating image quality based on the fidelity of perceived information, in line with human visual perception. By using a set of multifaceted no-reference metrics, we aim to ensure a robust and comprehensive evaluation of fusion performance. Additionally, in selecting no-reference evaluation metrics, we ensured that these metrics are widely recognized in the image fusion field to ensure their objectivity in assessing the quality and effectiveness of fused images.

#### 4.1.3. Detection Evaluation Details

An important objective of fusion tasks is to aid subsequent high-level vision tasks. However, the non-parametric evaluation indicators of fusion image quality may not always align perfectly with performance in high-level vision tasks, which can lead to an incomplete assessment of fusion results from the perspective of fusion tasks alone. Therefore, to further expound upon the efficacy of our proposed method in aiding high-level vision tasks, particularly in environments with harsh light interference, we opted to assess the performance of our model and comparative methods in the most representative high-level vision task, namely the detection task. Specifically, we opted to use the YOLOv5 [60] object detection algorithm to assess the effectiveness of fusion images in high-level vision tasks. We trained the detection network using a dataset comprising 500 manually annotated visible images infrared and images with key category labels for people and cars from the MFNet dataset. Subsequently, we conducted object detection on 40 normal background scenes selected from the MFNet to evaluate our model’s performance in normal background scenes and on 25 harsh light background scenes from the MFNet and 15 high-exposure scenes from the RoadScene dataset, totaling 40 special scenes, to evaluate our model’s performance in harsh light background scenes. For the quantitative evaluation of detection results, we chose F1_score, detection accuracy at 50% confidence (AP@50), detection precision at 70% confidence (AP@70), detection precision at 90% confidence (AP@90), and average detection accuracy from 50% to 95% confidence (AP@[50:95]) as evaluation parameters to appraise the quality of detection results.

### 4.2. Comparative Experiments on the TNO Dataset

To validate the performance of our proposed method in general scenes, we first selected 26 different scenes from the TNO dataset, a standard dataset comprising both visible and infrared images in regular scenarios, to qualitatively and quantitatively evaluate our model and comparative methods from the perspective of fusion tasks.

#### 4.2.1. Qualitative Comparison

**Figure 6:**
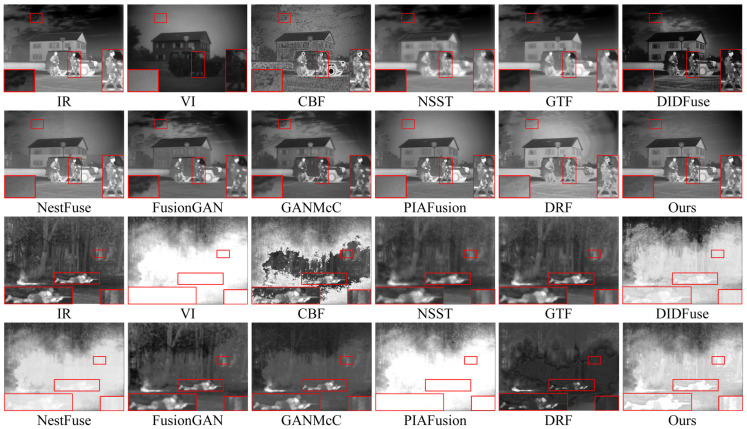
Qualitative results for the TNO dataset images. The images in the red boxes show texture details in the original images or magnified details of important targets.

The qualitative results of two typical image pairs from the TNO dataset are displayed in Figure 6. Notably, the red boxes in the figure are used to zoom in on key target objects and texture background regions within the images, providing a clearer perspective.

In the first scene of the presentation of the qualitative results, we used red boxes to select the prominent human target area and the sky cloud texture area. Regarding the fusion results of the prominent human target area, CBF showed the worst fusion performance, with the prominent human target being disturbed by a large amount of useless noise information, blurry edges, and significant feature loss; NSST, GTF, and FusionGAN methods severely blurred the edges and texture details of humans; DIDFuse, DRF, and GANMcCmethods preserved the overall significant information of human targets but still suffered from the problem of useless information interference caused by partial loss of significant features and weakening of the prominence of human targets. Only NestFuse, PIAFusion, and our method could better preserve the texture of human targets while avoiding the loss of significant information. Unfortunately, NestFuse and PIAFusion had serious flaws in handling the texture of infrared images, as shown in the sky cloud texture area, where the texture details were completely lost. Therefore, only our method could highlight prominent infrared target details while preserving intricate texture features.

The second scenario of the qualitative presentation is a typical challenging fusion scenario attributable to the wide-ranging smoke occlusion in the visible image. In the fusion results of this scene, CBF still introduced a large amount of useless noise; the human targets in the fusion results of NestFuse and PIAFusion were almost completely lost; the saliency of human targets in the DIDFuse method significantly decreased. Among the methods that highlighted the saliency of human targets, NSST, GTF, FusionGAN, GANMcC, and DRF all showed varying degrees of edge blurring of human targets. Only our method, due to the introduction of the edge guidance module, greatly emphasized the edge features of human targets, making them well-distinguished from the smoky background. Considering the performance of the two scenes together, only our method could better preserve richer texture details while enhancing the salient information of infrared targets.

#### 4.2.2. Quantitative Comparison

For a more objective assessment of our proposed method’s performance on the TNO dataset, we proceeded with additional quantitative evaluations of the method results. The quantitative assessment results of 26 image pairs in the TNO dataset are depicted in Table 1. Our proposed approach exhibits superior performance in terms of EN, MS-SSIM, PSNR, $FMI_{pixel}$, and $Q_{abf}$, ranking second in SD, and falling slightly behind NestFuse and PIAFusion in the VIF metric. These findings suggest that the HLEA module we designed does not entail sacrificing fusion performance in normal scenes entirely for the sake of harsh light special scenes. In summary, our approach retains more meaningful information strongly correlated with input images, thereby achieving the overall best performance qualitatively and objectively.

### 4.3. Comparative Experiments on the MFNet Dataset

Scenes in the TNO dataset are generally simple, with a lower number of objects included. To substantiate the efficacy of our proposed approach in more complex scenes, we further selected 25 scenes from the MFNet dataset for qualitative and quantitative evaluations of our model and alternative methods in terms of fusion tasks. Unlike the TNO dataset, where both visible and infrared spectra are represented in grayscale, the MFNet dataset consists of color images for visible. In the fusion process, initially, we transform the visible images from the RGB color space to the YCrCb color space. Then, we integrate the luminance channel Y with the infrared images. Afterward, we merge the fused image with the Cr and Cb channels of the visible images. Finally, the resultant image is reconverted to the RGB color space to derive the color fusion image.

**Figure 7:**
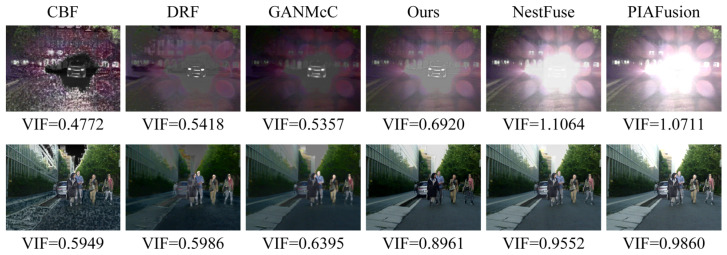
VIF metric results for fused images in normal and harsh light conditions. The first row and the second row correspond to the fusion results in harsh light and normal environments, respectively.

It is worth noting that although we selected a set of widely accepted no-reference metrics in the fusion field for multi-angle image evaluation, these metrics are generally effective only for evaluating fused images under normal conditions and have limitations in evaluating fused images under harsh lighting conditions. In Figure 7, we demonstrate this phenomenon by showing fused images under normal and harsh light conditions and using the no-reference metric VIF, which is consistent with human visual perception.

From the images, it can be seen that under normal conditions, the VIF metric for fused images aligns with human subjective perception. However, under harsh light conditions, the VIF metric for fused images with harsh light interference is higher, deviating from actual perception. Therefore, we adopt different evaluation methods for fused images under normal and harsh light conditions in the MFNet. For the former, we use a combination of conventional qualitative result presentation and quantitative no-reference metric estimation, while for the latter, we use a combination of qualitative result presentation and mean opinion score (MOS) image quality evaluation. By supplementing the MOS subjective evaluation of fused images under harsh light conditions, we can more intuitively and effectively demonstrate the superior performance of our method in image fusion under harsh lighting conditions.

#### 4.3.1. Qualitative Comparison

(1) Fusion Results Display

**Figure 8:**
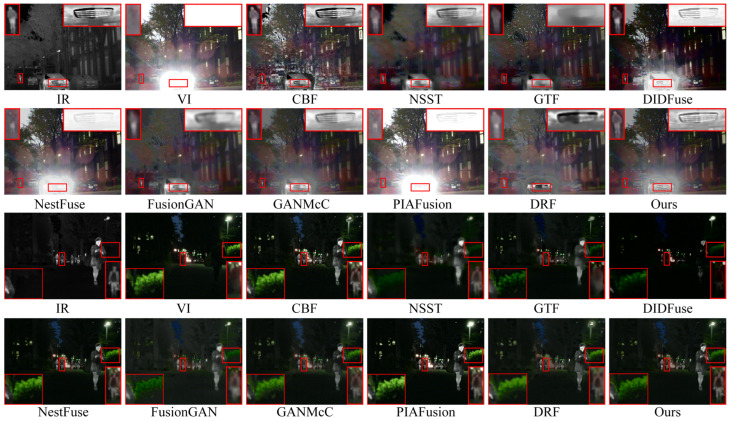
Qualitative results for the MFNet dataset images. The images in the red boxes show texture details in the original images or magnified details of important targets.

Figure 8 illustrates the intuitive qualitative results of two representative image pairs in the MFNet dataset. Scene one depicts daytime with harsh light interference, while scene two portrays nighttime under normal background conditions. The red boxes in the images zoom in on important target objects and textured background areas.

In the qualitative visual performance of the two typical scenes in Figure 7, the results generated by our proposed method noticeably outperform those generated by other methods. This is because, compared to most advanced fusion algorithms, our method can effectively suppress harsh light in the fusion results when there is harsh light interference in the scene, while maintaining the significance of targets susceptible to thermal radiation and the richness of texture details under harsh light occlusion. The capability of our method to handle harsh light interference is crucial, as the removal of harsh light is decisive for enhancing performance in subsequent high-level vision tasks such as object detection. Furthermore, in typical road scenes, our fusion method still visibly preserves a large amount of texture details while retaining sufficient thermal radiation information.

According to the qualitative results of image fusion in two typical scenes, we can classify the selected comparative methods into two groups based on their ability to remove harsh light interference. The first group includes DIDFuse, NestFuse, and PIAFusion, which cannot effectively remove harsh light information from the fusion results. For example, in scene one, the fusion results of NestFuse and PIAFusion are almost completely obscured by harsh light information, and a large amount of vehicle texture in DIDFuse is submerged in harsh light. The second group includes CBF, NSST, GTF, FusionGAN, GANMcC, and DRF, which have essentially removed harsh light from the fusion results. However, due to the lack of targeted research on harsh light removal, the thermal target texture details under harsh light occlusion in the fusion results are blurred to varying degrees. For example, in scene one, the vehicle texture under harsh light occlusion is most severe in methods such as NSST, GTF, FusionGAN, and DRF. Only our method, while removing harsh light interference, retains sufficient thermal radiation information and rich texture details. In typical scenes, only our method in the second group maintains the same fusion performance as NestFuse and PIAFusion, maintaining sufficient texture information and enhanced details during the fusion process, making the fused images appear more natural. In summary, our approach amalgamates the benefits of the aforementioned two methodologies, preserving rich texture details and thermal target prominence while avoiding harsh light background interference.

(2) MOS Subjective Quality Evaluate

The MOS is a subjective evaluation method employed to assess the quality of images, audio, or video. It represents the overall perceived quality of the media by summarizing ratings from several observers. We opted to employ this method to assess the quality of fused images in high-light conditions. In the experiment, we gathered the mean opinion scores from 15 reviewers for 40 fused images under high exposure or bright light conditions in five dimensions: image clarity, color fidelity, light resistance, artifact noise, and overall impression. The corresponding overall MOS was then calculated. Each dimension was rated on a discrete ordinal scale, with a total of 5 points corresponding to 5 levels. Scores from 1 to 5 indicate very poor, poor, average, good, and excellent quality for that dimension, respectively. The final MOS table is presented in Table 2.

In the subjective quality assessment of fused images under harsh light conditions, our method achieves optimal performance in image clarity, color fidelity, and overall impression, and suboptimal performance in light resistance and noise artifacts. Based on the average opinion scores across the five dimensions, the subjective visual performance of our method is the best among all methods. Furthermore, it is noteworthy that the best three methods for the light resistance metric are FusionGAN, our method, and GANMcC, while the worst are NestFuse and PIAFusion. This aligns with the harsh light removal performance in fused images under harsh light conditions as shown earlier, demonstrating that our method effectively mitigates the severe interference caused by harsh light for viewers.

#### 4.3.2. Quantitative Comparison

To impartially illustrate the efficacy of our suggested approach in complex scenes, we carried out quantitative non-reference evaluation index testing on a dataset consisting of 25 pairs of images from MFNet, and the findings are outlined in Table 3. It is important to note that, as previously mentioned, due to the limitations of non-parametric evaluation metrics, we only perform non-parametric evaluations on fused images of normal background scenes here. In Section 4.5, “High-level vision task-driven evaluation”, we quantitatively assess the performance of our model in scenarios with harsh light interference. From the results in Table 3, it is evident that in typical scenes, our approach achieves optimal performance concerning EN, MS-SSIM, $FMI_{pixel}$, and $Q_{abf}$, and the second best performance in terms of SD and VIF, while PIAFusion achieves the best performance in SD and VIF, and the second best performance in EN, MS-SSIM, $FMI_{pixel}$, and $Q_{abf}$. This indicates that while maintaining the same high level of fusion image visual naturalness as PIAFusion, our approach retains a greater amount of texture and edge information from the source image. In summary, our method attains the highest standard of objective evaluation, ensuring sufficient thermal radiation information while maintaining a significant amount of texture intricacies.

### 4.4. Comparative Experiments on the M3FD Dataset

It is commonly acknowledged that the ability of deep learning methods to generalize is also a crucial aspect of assessing the quality of their models. Given that our model underwent training on the MFNet-based MSRS dataset, to substantiate the generalization performance of our model, we further conducted qualitative and quantitative evaluation carried out on 30 pairs of images sourced from the M3FD dataset using our model and comparative methods. It is pertinent to mention that both the visible images in the M3FD and MFNet datasets are in color, so we obtained color fusion images on the M3FD dataset following the steps of image fusion in the MFNet dataset.

#### 4.4.1. Qualitative Comparison

**Figure 9:**
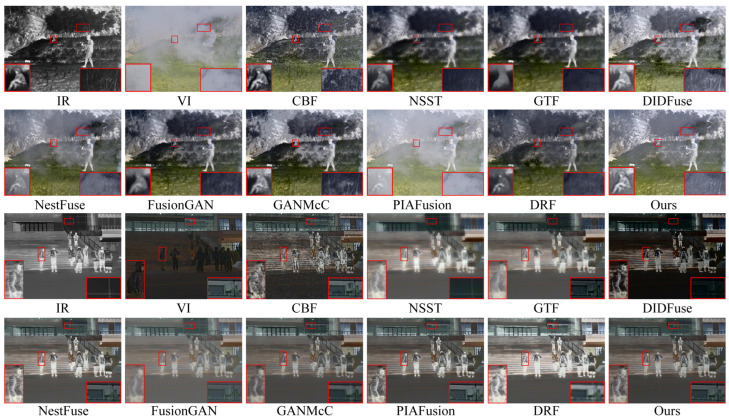
Qualitative results for the M3FD dataset images. The images in the red boxes show texture details in the original images or magnified details of important targets.

Figure 9 displays the intuitive qualitative outcomes of two representative image pairs sourced from the M3FD dataset. The highlighted red boxes within the figure provide a closer look at crucial target objects and textured background regions present in the images.

In the first scene of the qualitative results display, due to the occlusion of smoke in the visible image, meaningful salient targets and texture details are almost entirely concentrated in the infrared image. This poses a serious problem for algorithms that prioritize infrared image salient object pixel intensity and clear visible image target texture details as a prior assumption for image fusion. From the fusion results, the texture details in the branch areas of CBF, NSST, GTF, NestFuse, and PIAFusion are almost completely lost or obscured by smoke, and there are varying degrees of loss in the texture of branch areas in FusionGAN, GANMcC, and DRF as well. Correspondingly, the prominence of infrared targets in the fusion outcomes of these algorithms is also weakened to varying degrees. Only DIDFusion and our method can uphold the prominence of infrared thermal targets while preserving the complete texture details of the branch areas. However, compared to the fusion results of DIDFusion, our fusion outcomes exhibit a more natural appearance and demonstrate improved consistency with human visual perception.

Within the second scenario of the qualitative results display, we set two regions of interest, one is the area of human targets and clothing texture, and the other is the area of glass texture. Across the fusion outcomes produced by various methods, although CBF, GTF, DIDFuse, FusionGAN, GANMcC, and DRF all retained the basic saliency of human targets, the texture details of human clothing appeared significantly blurred. Only NSST, NestFuse, PIAFusion, and our method retained the texture details of clothing completely, but NSST sacrifices visible light image texture for the retention of infrared image clothing texture details. In NSST, the texture area behind the glass is completely lost, indicating severe loss of visible image information in the fusion process. Therefore, only NestFuse, PIAFusion, and our method excel in the preservation of the texture details of both the infrared and visible images in this scene. Unfortunately, NestFuse and PIAFusion performed poorly in the smoke interference scene in scene one. In summary, considering the performance in both scenes, our method uniquely excels in enhancing the saliency of infrared targets while simultaneously preserving richer texture details.

#### 4.4.2. Quantitative Comparison

We further conducted quantitative estimation experiments on fusion methods in M3FD, and the outcomes are depicted in Table 4. In the M3FD generalization dataset scene, our approach attains the highest performance in MS-SSIM and $Q_{abf}$ and the runner-up best performance in VIF, $FMI_{pixel}$, and PSNR. This indicates that our method still retains sufficient source image texture and edge information and has good visual fidelity on non-training datasets, achieving the best overall level with good generalization performance.

### 4.5. High-Level Vision Task-Driven Evaluation

Fusion tasks should not only improve the depth of information encapsulated in the fused image scenes but also assist in high-level vision tasks. Therefore, we evaluated qualitatively and quantitatively assessments of the fusion outcomes achieved by our method as well as other methods in the high-level vision task of target detection to demonstrate the effectiveness and superiority of our method in assisting high-level vision tasks, especially in scenes with conflicting harsh light interference.

#### 4.5.1. Quantitative Comparison

(1) Harsh light scene detection results

Quantitative evaluation results of detection in harsh light and high-exposure scenes are shown in Table 5. From the outcomes, it becomes apparent that in harsh light special scenes, the infrared image achieves the best level of detection accuracy in terms of F1 score and 50% confidence level. This indicates that the infrared image information is indispensable for improving the detection performance of fusion results in harsh light environments, and our method effectively utilizes the thermal radiation target information in the fusion process, achieving detection performance similar to that of the infrared image in terms of F1_Score and Ap@50 metrics. Among fusion algorithms capable of removing harsh light interference, NSST and FusionGAN can also maintain basic detection accuracy performance at low confidence levels. However, due to the loss of thermal radiation target edges and crucial textural intricacies evident in the fusion outcomes, the detection performance decreases at high confidence levels and average confidence levels. Additionally, compared to GANMcC, which can effectively remove harsh light and include basic texture details, our method shows better overall performance in detection accuracy at different confidence levels, indicating that our method contains richer semantic information in the fusion results. It is worth noting that two fusion algorithms, NestFuse and PIAFusion, which cannot remove harsh light interference, both fail to achieve satisfactory detection results in harsh light interference scenes.

(2) Normal light scene detection results

Quantitative evaluation results of detection in normal background are shown in Table 6. From the results, it can be seen that algorithms such as NSST, DIDFuse, FusionGAN, and GANMcC, which perform well under harsh light interference, fail to reach top-tier detection performance. However, due to our algorithm’s emphasis on the richness of semantic information in fusion results and the performance of assisting advanced visual tasks, it still maintains a leading position in detection results at high confidence levels and average confidence levels. In conclusion, our proposed fusion method can effectively assist detection tasks under harsh light interference while ensuring high-level detection results in normal background environments.

#### 4.5.2. Qualitative Presentation

**Figure 10:**
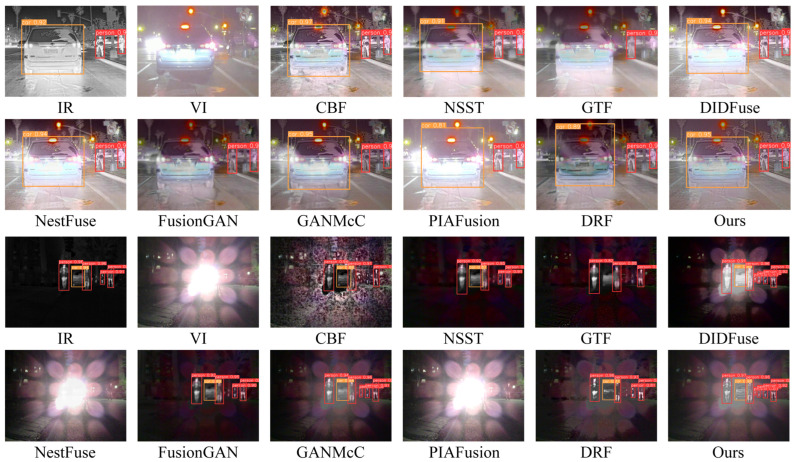
Qualitative results of target detection in harsh light and high exposure scenes for infrared images, visible images, and fusion images generated by various algorithms. Each pair of lines represents the detection results for one scene.

**Figure 11:**
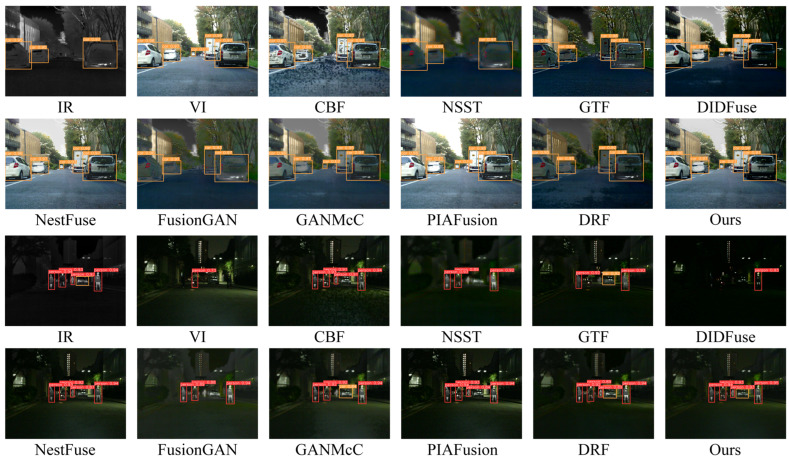
Qualitative results of target detection in normal light scenes for infrared images, visible images, and fusion images generated by various algorithms. Each pair of lines represents the detection results for one scene.

Furthermore, in Figure 10 and Figure 11, we provide visual detection examples in four scenarios: two in harsh light and overexposure environments and two in normal environments, demonstrating the intuitive merits of our fusion algorithm in promoting target detection. In the first scenario of Figure 9, PIAFusion exhibits deviation in detection bounding boxes due to overexposure interference, while among the methods capable of removing harsh light interference, GTF and FusionGAN exhibit vehicle and pedestrian omissions due to the blurriness of target edges and textures in fusion results, and DRF generates detection box deviations. Among all fusion methods, only CBF, NSST, DID-Fuse, NestFuse, GANMcC, and our method complete the target detection task comprehensively. In the more adverse lighting conditions of the second scenario in Figure 9, NestFuse fails to detect any targets due to its inability to remove harsh light interference, and CBF, NSST, and GANMcC suffer from target omissions due to the loss of semantic information during fusion. Only DIDFuse and our method complete the target detection comprehensively. However, regrettably, DIDFuse’s excellent performance under harsh light comes at the cost of sacrificing normal environment detection performance. In the second nighttime scenario of Figure 10, DIDFuse’s fusion result loses almost all thermal target information, resulting in severe omissions, while our result maintains good detection performance comparable to NestFuse and PIAFusion. This indicates that our HLEA module in fusion results does not blindly remove harsh light by favoring infrared images nor does it ignore its detection performance in normal scenes.

### 4.6. Module Effectiveness Validation

#### 4.6.1. Edge Feature Extractor Effectiveness Analysis

**Figure 12:**
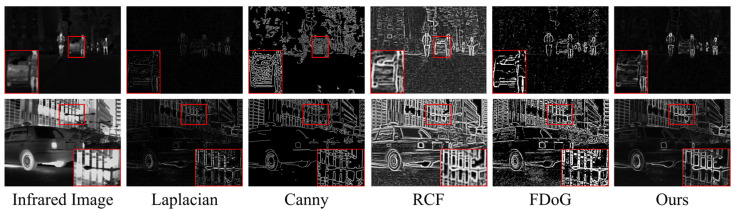
Results of edge feature detection using different algorithms. The images in the red boxes show texture details in the original images or magnified details of important targets.

In comparison to other edge extraction algorithms, our edge feature extractor is specifically tailored for the subsequent feature fusion in the fusion network, ensuring that the extracted edge features better meet the needs of the following modules. To illustrate the effectiveness and specificity of our design, we compared the edge images decoded from the edge features obtained by the final EFEB block of our feature extractor with those from four different image edge extraction algorithms. The four selected algorithms are Laplacian edge extraction [61], Canny edge extraction [62], RCF edge extraction [63], and FDoG edge extraction [64]. The comparison of edge images in two typical scenarios is presented in Figure 12.

From the overall edge results, our method demonstrates significant advantages in maintaining edge coherence and noise reduction clarity, although it is not as strong in edge line pixel intensity as DRF and FDoG. This result aligns perfectly with the fusion task’s requirements. In the subsequent feature fusion process, we use edge features to guide the fusion, whereas the excessive noise in DRF and FDoG could seriously interfere with the accuracy of feature fusion. Furthermore, overly thick edge lines may lead to artifacts at object edges in the fused image, thus lowering the image quality. Additionally, our edge feature extractor is designed with three EFEB modules, each extracting edge features at different levels, to guide feature fusion from shallow to deep layers. This specific design makes our method more suitable for subsequent fusion tasks compared to other edge extraction algorithms.

#### 4.6.2. HLEA Module Effectiveness Analysis

**Figure 13:**
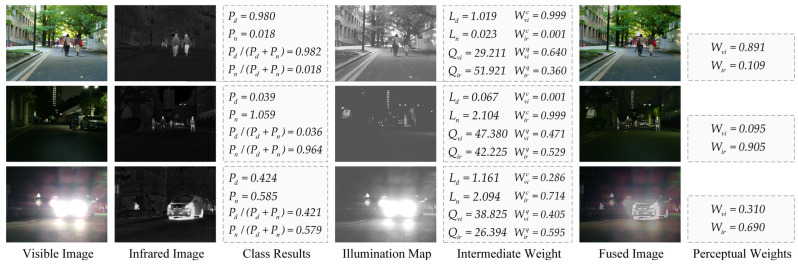
The intermediate and final results of the HLEA module under three typical scenarios in normal and harsh light environments are displayed. The “Class Results” column shows the illumination classification results, “Intermediate Weight” presents the illumination decomposition and image quality estimation results, and “Perceptual Weight” displays the final output results.

To more effectively demonstrate the rationale and validity of the illumination classification, illumination decomposition, and image quality estimation blocks within our HLEA Figure 13 design, we selected three representative scenarios under both normal and harsh light conditions for detailed presentation. These scenarios allow us to demonstrate the performance of the HLEA module in practical applications and thoroughly analyze its intermediate calculations and final outcomes under varying lighting conditions. Specifically, Figure 13 presents the detailed application of the HLEA module in these scenarios, including illumination classification results, illumination decomposition effects, image quality estimation results, intermediate weighting results, and the final weighting results.

The first and second rows in the figure display the daytime and nighttime scenarios under normal conditions, respectively. In these two scenarios, the illumination classification network successfully categorizes the scenes as either daytime or nighttime. On this basis, the illumination maps generated by the illumination decomposition network have little effect on the weights calculated from the classification results. However, the values of $W_{vi}^{c}$ and $W_{ir}^{c}$ behave too extremely in this situation, so correction is required. The corrected $W_{vi}^{q}$ and $W_{ir}^{q}$ values, calculated by the image quality estimator, achieve a balanced adjustment between the infrared and visible components in the final weights while maintaining the inclination of the classification and decomposition network’s weight preferences.

The third row in the figure depicts a harsh light environment scenario. In this scenario, the category probabilities calculated by the illumination classification network show bias, where the weight for the visible component is too high, and the weight for the infrared component is too low. After adjustment by the illumination map, the weight of the visible component was effectively suppressed, and the weight of the infrared component was increased. Subsequently, after correction by the image quality estimator, the final perceptual weight for the infrared component became significantly higher than the visible light component, aligning with our expected results. Combining the intermediate and final results across the three typical scenarios, our designed HLEA performs as expected under normal conditions.

#### 4.6.3. Ablation Experiments

**Figure 14:**
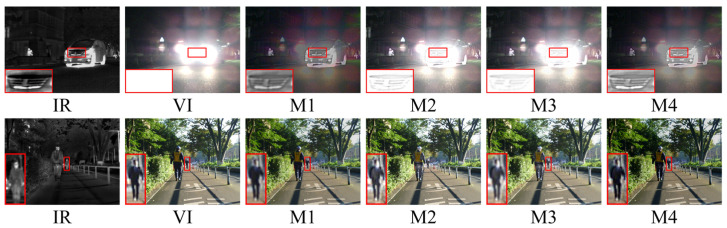
Module corresponding ablation experiments. The images in the red boxes show texture details in the original images or magnified details of important targets.

In our work, we have designed multiple modules that can be categorized into two parts based on their functionalities. The HSIF and EFEB modules in edge feature extraction, along with the EGHF module in feature fusion, seek to enhance edge characteristics and surface texture details of thermal radiation targets, thereby enhancing the semantic richness of the fusion results. The figuration of HLEA primarily seeks to mitigate harsh light interference and achieve anti-harsh light interference fusion for the algorithm. To demonstrate the effectiveness of these two parts of module design, we conducted targeted ablation experiments on the two parts of modules. Specifically, the model that removes the modules related to rich semantic information but includes the HLEA module is denoted as M1, and the model that includes the modules related to rich semantic information but lacks the HLEA module is denoted as M2. The model that removes both parts of modules is denoted as M3, and our original model is represented as M4. The quantitative and qualitative findings from the experiments are illustrated in Figure 14 and Table 7.

## 5. Conclusions

In order to solve the impact of harsh light interference on the quality of fused images in nighttime road environments, which leads to a sharp decline in fusion algorithm performance, we propose an image fusion network that effectively copes with harsh light interference. The edge feature extraction module optimizes information entropy to retain key features. The HLEA module addresses the degradation in fusion quality caused by the conflict between low information entropy and high pixel values in image-harsh regions. And the EGHF module achieves robust feature fusion by minimizing irrelevant noise entropy and maximizing useful information entropy. The experimental outcomes reveal that compared with the existing advanced fusion algorithms, the proposed algorithm has significant advantages in the quality of fusion images and the performance of subsequent advanced visual tasks, effectively solving the influence of harsh light interference on image fusion performance in the night road environment, and provides harsh technical support for composite high-level vision tasks, such as night vehicle assisted driving.

## Figures and Tables

**Table 1:** Quantitative results on the TNO dataset for selected comparative approaches. The results in red represent the best outcome, and those that are blue indicate suboptimal results.

	EN	SD	MS-SSIM	VIF	PSNR	*FMI_pixel_*	*Q* *_abf_*
CBF	7.1291	40.4001	0.7084	0.5895	62.9445	0.8646	0.4200
NSST	6.8753	42.4164	0.8237	0.6565	61.7691	0.8921	0.2986
GTF	6.8811	42.7918	0.8238	0.6527	61.7706	0.8897	0.4202
DIDFuse	7.2034	52.4016	0.8796	0.8140	61.9209	0.8645	0.3419
NestFuse	7.1964	44.6529	0.8825	0.9437	62.6227	0.8911	0.5273
FusionGAN	6.5960	30.9010	0.7392	0.6125	60.9479	0.8709	0.2252
GANMcC	6.8340	34.1770	0.8375	0.6736	61.7864	0.8816	0.2646
PIAFusion	6.9998	44.5875	0.8655	0.9345	61.6819	0.8949	0.4854
DRF	6.7745	33.8643	0.7115	0.5633	61.3385	0.8766	0.1977
Ours	7.2156	44.8749	0.9418	0.8696	62.9953	0.8954	0.5467

**Table 2:** Mean opinion scores for fused images under harsh light conditions. Each dimension is rated from 1 to 5, with higher scores signifying excellent performance in that dimension and lower scores indicating the opposite. The results in red represent the best outcome, and those that are blue indicate suboptimal results.

	Image Clarity	Color Fidelity	Light Resistance	Artifact Noise	Overall Impression	Average Score
CBF	2.0567	1.9917	3.0733	1.6833	2.1033	2.1817
NSST	1.6483	1.8933	3.3917	2.5183	2.0950	2.3093
GTF	2.0133	2.4983	3.2833	2.2317	2.1450	2.4343
DIDFuse	2.4717	2.2650	3.0267	2.5833	2.3283	2.5350
NestFuse	3.1967	3.2783	2.5433	3.4917	3.0367	3.1093
FusionGAN	1.7517	2.3583	3.6433	2.0250	2.1433	2.3843
GANMcC	2.8683	2.8417	3.4017	2.9200	2.9917	3.0047
PIAFusion	2.7567	2.5933	2.2239	3.0733	2.4217	2.6138
DRF	1.7567	2.0967	3.1983	1.7533	1.8833	2.1377
Ours	3.3233	3.3275	3.5200	3.3833	3.4317	3.3918

**Table 3:** Quantitative results on the MFNet dataset for selected comparative approaches. The results in red represent the best outcome, and those that are blue indicate suboptimal results.

	EN	SD	MS-SSIM	VIF	PSNR	*FMI_pixel_*	*Q_abf_*
CBF	6.5949	36.8343	0.7942	0.7370	64.4616	0.9031	0.5697
NSST	5.8504	23.7295	0.6489	0.6522	63.7954	0.9224	0.2350
GTF	5.8667	23.7152	0.8471	0.5788	64.1360	0.9101	0.4076
DIDFuse	4.8717	39.6267	0.8087	0.4136	63.6994	0.9075	0.2305
NestFuse	6.6019	44.1226	0.9533	0.9247	65.0529	0.9241	0.6275
FusionGAN	5.6645	20.4431	0.7226	0.5559	64.3055	0.9056	0.1476
GANMcC	6.1798	29.4363	0.8767	0.6752	66.0370	0.9088	0.2923
PIAFusion	6.6460	49.8703	0.9549	1.0224	63.6291	0.9255	0.6550
DRF	5.9020	22.6209	0.7820	0.5675	65.2373	0.9060	0.1532
Ours	6.6945	45.2648	0.9697	0.9380	64.3400	0.9259	0.6750

**Table 4:** Quantitative results on the M3FD dataset for selected comparative approaches. The results in red represent the best outcome, and those that are blue indicate suboptimal results.

	EN	SD	MS-SSIM	VIF	PSNR	*FMI_pixel_*	*Q* *_abf_*
CBF	7.0005	35.4986	0.7125	0.7448	64.8020	0.8804	0.5605
NSST	7.1781	46.2808	0.7434	0.6730	63.0551	0.9050	0.1706
GTF	7.1995	45.8126	0.9080	0.7047	63.5330	0.8990	0.5161
DIDFuse	6.8437	46.5106	0.9270	0.8562	62.3610	0.8846	0.4565
NestFuse	6.8604	36.4310	0.9157	0.9030	64.3610	0.8969	0.4943
FusionGAN	6.4800	27.3050	0.8293	0.5518	63.8132	0.8842	0.2826
GANMcC	6.6894	32.2526	0.8978	0.7379	64.3309	0.8930	0.3318
PIAFusion	6.8480	36.0857	0.9242	0.9506	63.5715	0.9081	0.6104
DRF	6.8879	39.2190	0.7959	0.7132	62.6709	0.8824	0.1857
Ours	6.8937	36.5702	0.9611	0.9047	64.3942	0.9055	0.6364

**Table 5:** Quantitative target detection results of infrared images, visible images, and fusion images obtained by various algorithms in scenes with harsh light backgrounds in the MFNet and RoadScene datasets. The red, blue, and green denote the best, second, and third values.

Metrics		IR	VI	CBF	NSST	GTF	DIDFuse	NestFuse	FusionGAN	GANMcC	PIAFusion	DRF	Ours
F1_Score	people	0.811	0.372	0.695	0.770	0.554	0.678	0.643	0.709	0.857	0.729	0.724	0.811
	car	0.753	0.207	0.555	0.738	0.466	0.600	0.381	0.675	0.622	0.328	0.568	0.723
	all	0.782	0.290	0.625	0.754	0.510	0.639	0.512	0.692	0.739	0.528	0.646	0.767
AP@50	people	0.878	0.594	0.753	0.818	0.696	0.732	0.713	0.770	0.869	0.772	0.770	0.864
	car	0.791	0.493	0.682	0.781	0.580	0.663	0.618	0.743	0.725	0.598	0.678	0.778
	all	0.835	0.544	0.718	0.800	0.638	0.698	0.666	0.757	0.797	0.685	0.724	0.821
AP@70	people	0.770	0.488	0.721	0.776	0.654	0.708	0.700	0.697	0.779	0.705	0.695	0.808
	car	0.751	0.332	0.623	0.706	0.550	0.663	0.618	0.689	0.697	0.598	0.647	0.757
	all	0.761	0.410	0.672	0.741	0.602	0.686	0.659	0.693	0.738	0.652	0.671	0.783
AP@90	people	0.108	0.037	0.133	0.083	0.085	0.133	0.118	0.093	0.223	0.107	0.104	0.211
	car	0.221	0.000	0.257	0.068	0.056	0.352	0.383	0.078	0.383	0.363	0.233	0.453
	all	0.165	0.019	0.195	0.076	0.071	0.243	0.251	0.086	0.303	0.235	0.169	0.332
AP@[50–90]	people	0.599	0.351	0.536	0.562	0.480	0.516	0.510	0.515	0.610	0.515	0.539	0.631
	car	0.606	0.247	0.527	0.547	0.424	0.599	0.533	0.535	0.595	0.487	0.511	0.688
	all	0.603	0.299	0.532	0.555	0.452	0.558	0.522	0.525	0.603	0.501	0.525	0.660

**Table 6:** Quantitative target detection results of infrared images, visible images, and fusion images obtained by various algorithms in scenes with normal backgrounds in the MFNet dataset. The red, blue, and green denote the best, second, and third values.

Metrics		IR	VI	CBF	NSST	GTF	DIDFuse	NestFuse	FusionGAN	GANMcC	PIAFusion	DRF	Ours
F1_Score	people	0.920	0.648	0.892	0.858	0.706	0.639	0.898	0.836	0.912	0.882	0.894	0.912
	car	0.693	0.886	0.847	0.681	0.857	0.826	0.888	0.857	0.903	0.874	0.807	0.890
	all	0.806	0.767	0.870	0.769	0.781	0.733	0.893	0.847	0.907	0.878	0.851	0.901
AP@50	people	0.964	0.717	0.917	0.890	0.752	0.707	0.936	0.871	0.935	0.916	0.936	0.956
	car	0.723	0.945	0.866	0.735	0.896	0.874	0.924	0.859	0.921	0.907	0.840	0.927
	all	0.844	0.831	0.892	0.813	0.824	0.791	0.930	0.865	0.928	0.912	0.888	0.942
AP@70	people	0.786	0.634	0.840	0.812	0.690	0.673	0.884	0.731	0.879	0.867	0.856	0.896
	car	0.723	0.903	0.866	0.735	0.844	0.874	0.885	0.859	0.861	0.871	0.840	0.891
	all	0.755	0.769	0.853	0.774	0.767	0.774	0.885	0.795	0.870	0.869	0.848	0.894
AP@90	people	0.070	0.095	0.183	0.122	0.148	0.119	0.134	0.122	0.139	0.157	0.109	0.159
	car	0.293	0.434	0.473	0.116	0.402	0.239	0.523	0.464	0.498	0.441	0.420	0.517
	all	0.182	0.264	0.328	0.119	0.275	0.179	0.329	0.293	0.319	0.299	0.265	0.338
AP@[50–90]	people	0.604	0.481	0.656	0.609	0.537	0.511	0.662	0.583	0.652	0.651	0.635	0.681
	car	0.556	0.750	0.727	0.557	0.708	0.695	0.760	0.711	0.743	0.741	0.682	0.767
	all	0.580	0.616	0.692	0.583	0.623	0.603	0.711	0.647	0.698	0.696	0.659	0.724

**Table 7:** The quantitative analysis for the average value of three fusion metrics, i.e., F1_score, mAP@50, and mAP@[50:95] on MFNet and RoadScene dataset with the ablation studies of our methods. The red and blue denote the best and second value.

Module		M1	M2	M3	M4
EFEB+HSIF+EGHF		✗	✓	✗	✓
HLEA		✓	✗	✗	✓
F1_Score	harsh light scenes	0.784	0.668	0.638	0.767
	normal light scenes	0.880	0.900	0.866	0.901
mAP@50	harsh light scenes	0.834	0.735	0.733	0.821
	normal light scenes	0.929	0.928	0.905	0.942
mAP@[50:95]	harsh light scenes	0.641	0.560	0.559	0.660
	normal light scenes	0.694	0.699	0.699	0.724

## Data Availability

Data are contained within the article.
